# Supplementary figures and images for: Species-Level Resolution of Female Bladder Microbiota from 16S rRNA Amplicon Sequencing
Source: mSystems. 2021 Sep 14;6(5):e00518-21. doi: 10.1128/mSystems.00518-21 (PMC8547459; doi:10.1128/mSystems.00518-21)

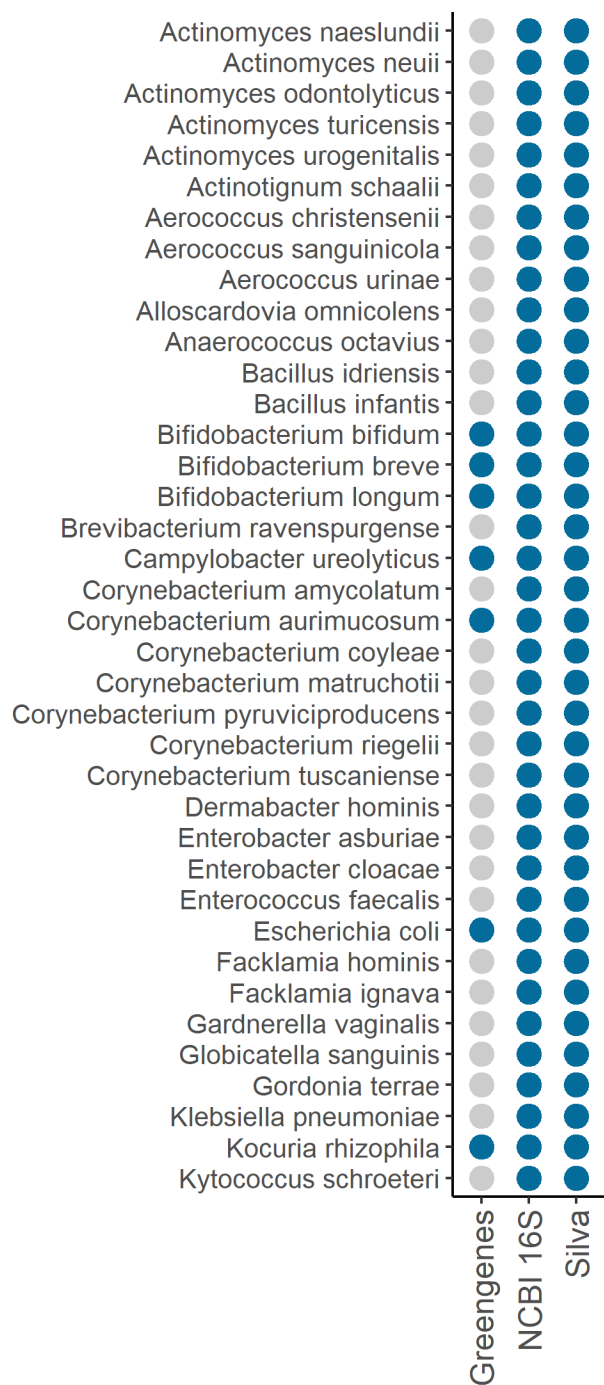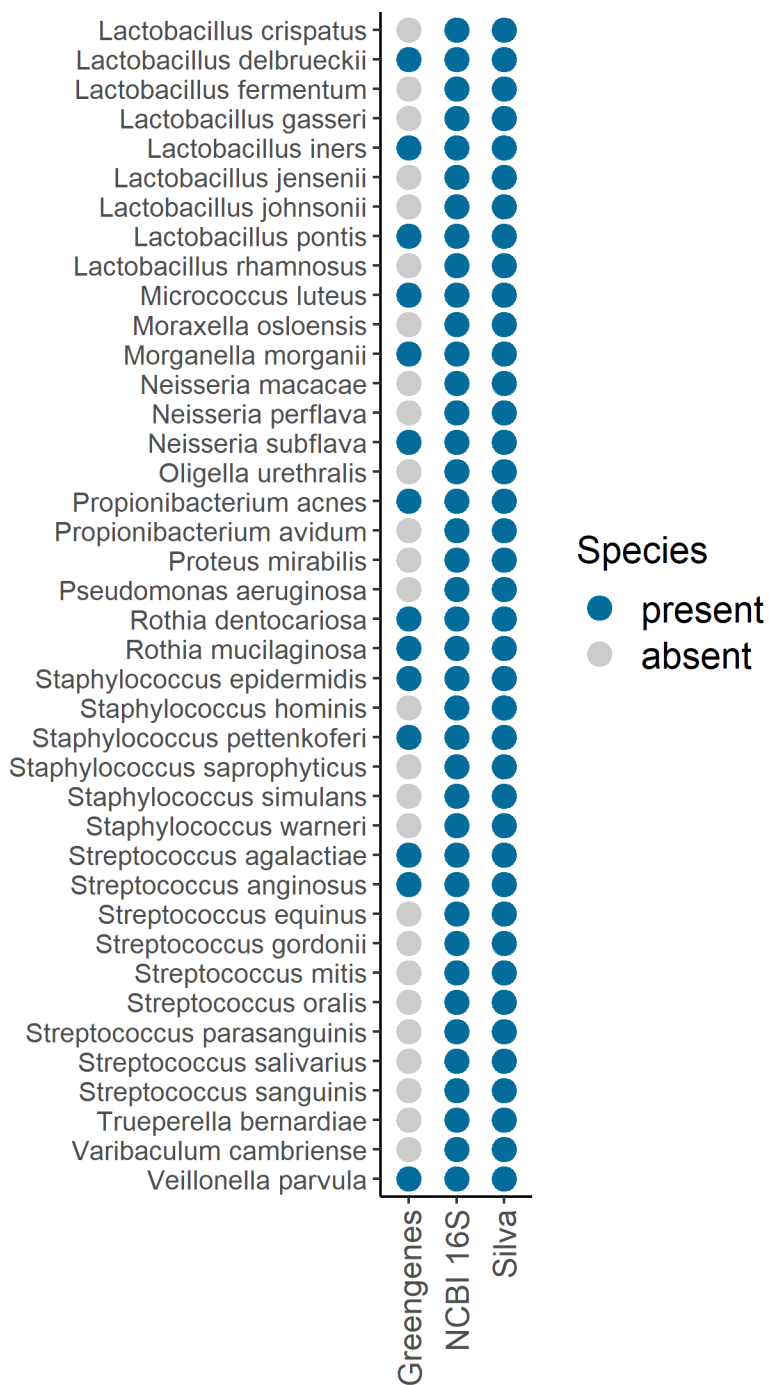

Species

● present

● absent

Supplement: FIG S1 [file msystems.00518-21-sf001.pdf]

A

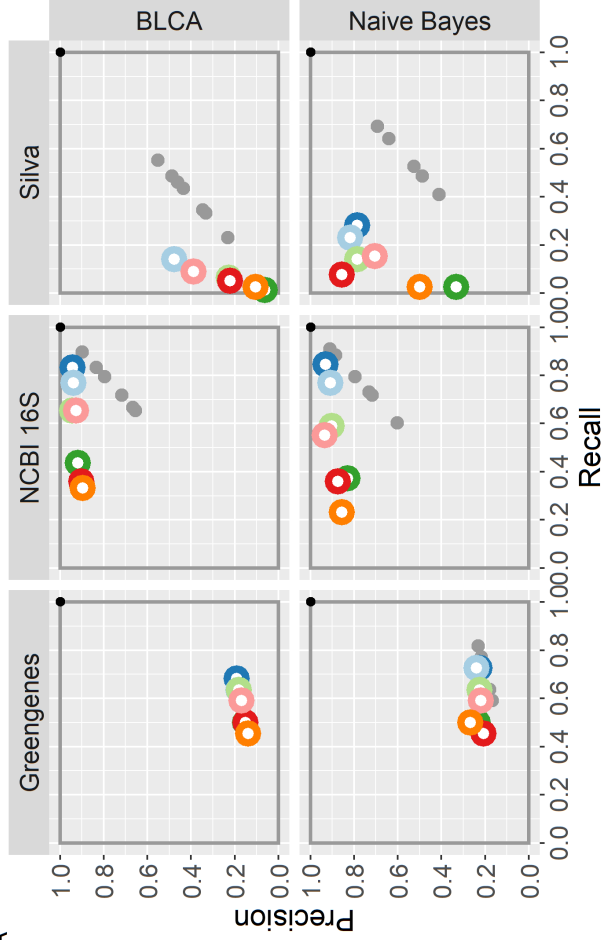

B

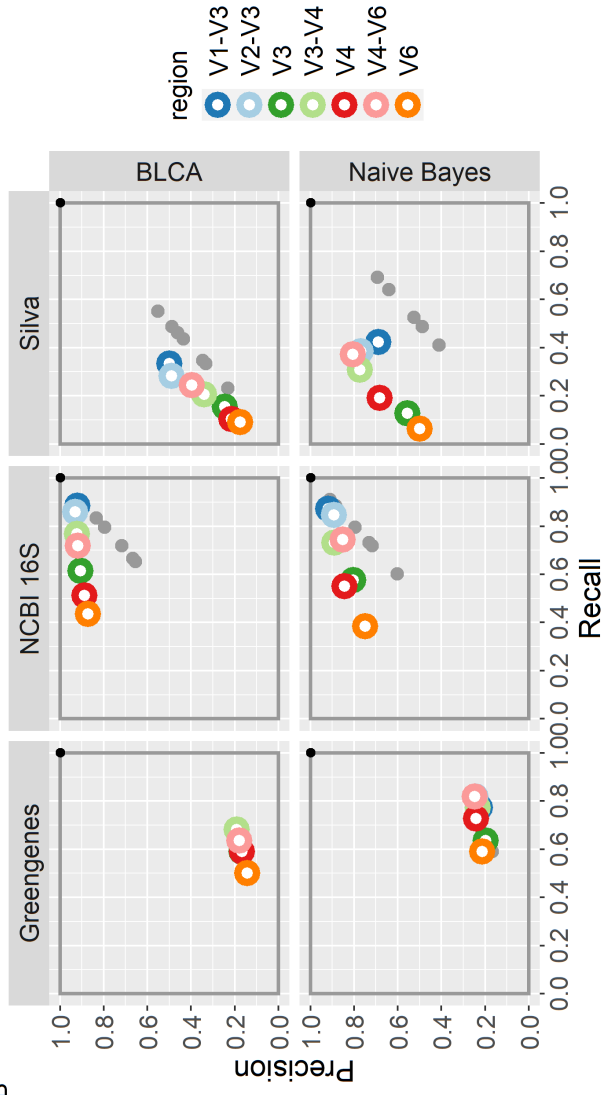

Supplement: FIG S2 [file msystems.00518-21-sf002.pdf]

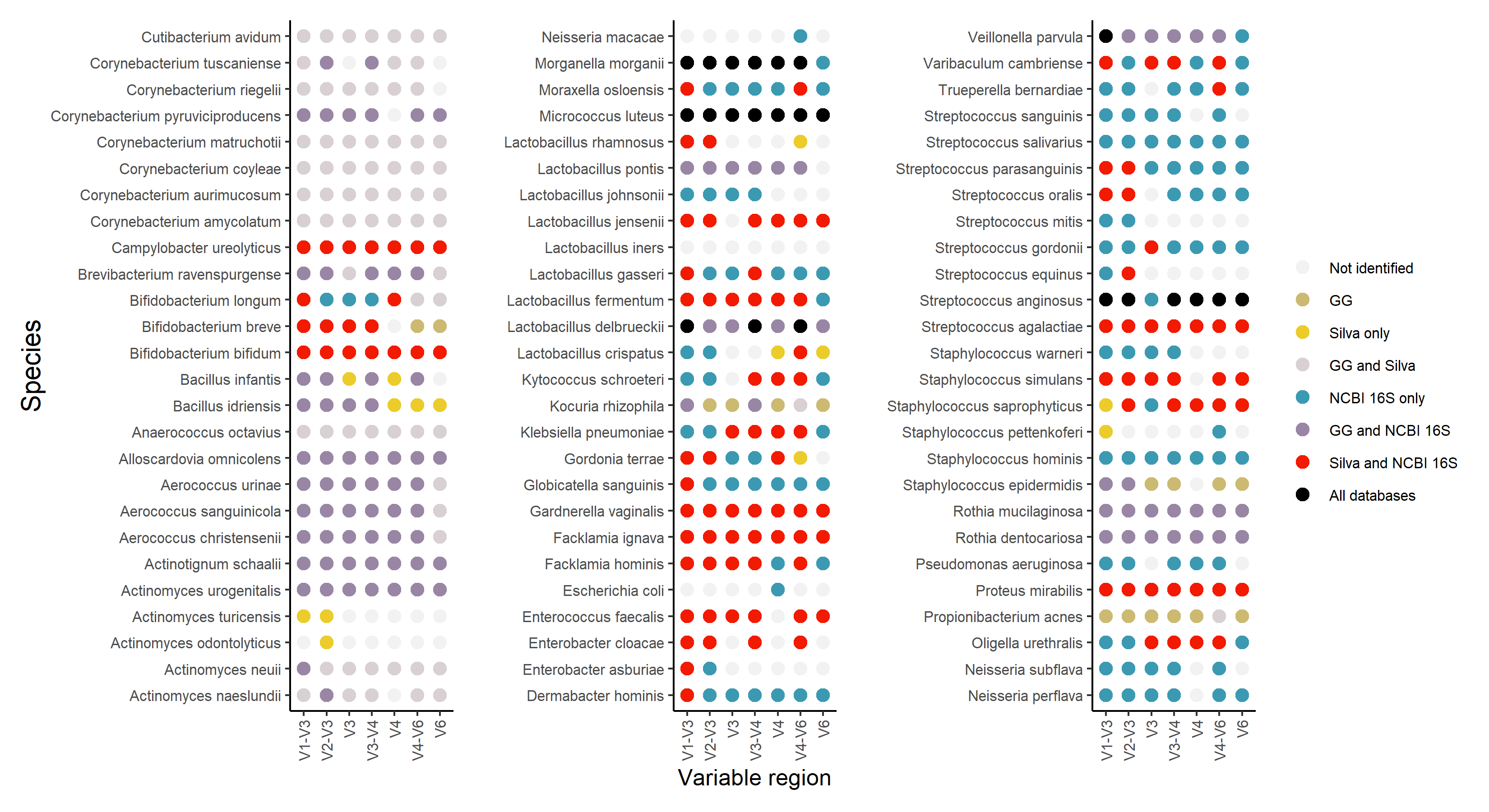

Supplement: FIG S3 [file msystems.00518-21-sf003.tiff]

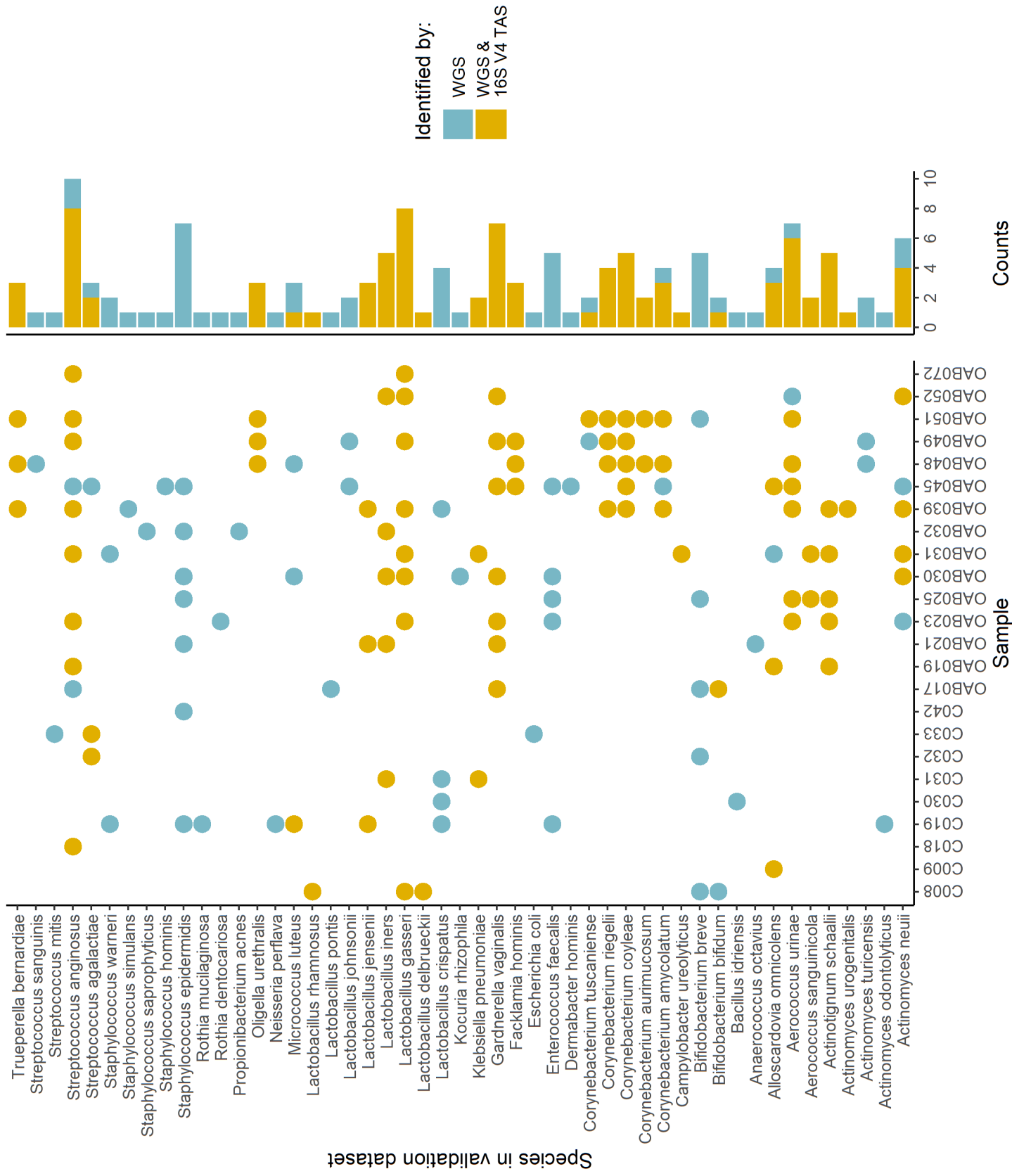

Supplement: FIG S4 [file msystems.00518-21-sf004.pdf]
