# Supplementary material for: Species-Level Resolution of Female Bladder Microbiota from 16S rRNA Amplicon Sequencing
Source: mSystems. 2021 Sep 14;6(5):e00518-21. doi: 10.1128/mSystems.00518-21 (PMC8547459; doi:10.1128/mSystems.00518-21)
Supplement: TABLE S1 [file msystems.00518-21-st001.docx]

**Table S1.** Names of bacterial type strains and accession numbers comprising the Thomas-White dataset downloaded from Silva. Species names from Supplemental Data 1 of Thomas-White et al 2018(1).

| species | type strain accession |
| --- | --- |
| Actinobaculum schaalii | FJ960443 |
| Actinomyces naeslundii | AB618790 |
| Actinomyces neuii | AM084229 |
| Actinomyces odontolyticus | AB818950 |
| Actinomyces turicensis | X78720 |
| Actinomyces urogenitalis | ACFH01000038 |
| Aerococcus christensenii | Y17005 |
| Aerococcus sanguinicola | AJ276512 |
| Aerococcus urinae | M77819 |
| Alloscardovia omnicolens | AM419460 |
| Anaerococcus octavius | Y07841 |
| Bacillus idriensis | AY904033 |
| Bacillus infantis | AY904032 |
| Bifidobacterium bifidum | S83624 |
| Bifidobacterium breve | AB006658 |
| Bifidobacterium longum | AP010889 |
| Brevibacterium massiliense | EU086793 |
| Campylobacter ureolyticus | ARGD01000016 |
| Corynebacterium amycolatum | X84244 |
| Corynebacterium aurimucosum | AJ309207 |
| Corynebacterium coyleae | X96497 |
| Corynebacterium matruchotii | ACSH02000003 |
| Corynebacterium pyruviciproducens | ATBY01000001 |
| Corynebacterium riegelii | Y14651 |
| Corynebacterium sp. | . |
| Corynebacterium tuscaniense | AY677186 |
| Dermabacter hominis | X91034 |
| Enterobacter asburiae | AB004744 |
| Enterobacter cloacae | CP001918 |
| Enterococcus faecalis | AB012212 |
| Escherichia coli | EU014689 |
| Facklamia hominis | AGZD01000007 |
| Facklamia ignava | AGZE01000005 |
| Gardnerella vaginalis | M58744 |
| Globicatella sanguinis | AB680901 |
| Gordonia terrae | AB920570 |
| Klebsiella pneumoniae | X87276 |
| Kocuria rhizophila | Y16264 |
| Kytococcus schroeteri | AJ297722 |
| Lactobacillus crispatus | AB008206 |
| Lactobacillus delbrueckii | AB007908 |
| Lactobacillus fermentum | AJ575812 |
| Lactobacillus gasseri | AB008209 |
| Lactobacillus iners | HE573916 |
| Lactobacillus jensenii | AF243176 |
| Lactobacillus johnsonii | HE573915 |
| Lactobacillus pontis | X76329 |
| Lactobacillus rhamnosus | AB008211 |
| Micrococcus luteus | CP001628 |
| Moraxella osloensis | FR726160 |
| Morganella morganii | AJ301681 |
| Neisseria macacae | HF558383 |
| Neisseria perflava | HF558366 |
| Neisseria subflava | AJ239291 |
| Oligella urethralis | AF133538 |
| Propionibacterium acnes | CP006032 |
| Propionibacterium avidum | AGBA01000019 |
| Proteus mirabilis | AJ301682 |
| Pseudomonas aeruginosa | HE978271 |
| Rothia dentocariosa | CP002280 |
| Rothia mucilaginosa | X87758 |
| Staphylococcus epidermidis | AB681292 |
| Staphylococcus hominis | X66101 |
| Staphylococcus pettenkoferi | AF322002 |
| Staphylococcus saprophyticus | AP008934 |
| Staphylococcus simulans | D83373 |
| Staphylococcus warneri | L37603 |
| Streptococcus agalactiae | AB023574 |
| Streptococcus anginosus | AFIM01000033 |
| Streptococcus equinus | AB680295 |
| Streptococcus gordonii | AF003931 |
| Streptococcus mitis | AF003929 |
| Streptococcus oralis | ADMV01000001 |
| Streptococcus parasanguinis | AF003933 |
| Streptococcus salivarius | AY188352 |
| Streptococcus sanguinis | AF003928 |
| Trueperella bernardiae | X79224 |
| Varibaculum cambriense | AJ428402 |
| Veillonella parvula | AB538437 |
|  |  |

Reference

1. Thomas-White K, Forster SC, Kumar N, Kuiken MV, Putonti C, Stares MD, et al. Culturing of female bladder bacteria reveals an interconnected urogenital microbiota. Nat Commun. 2018 Apr 19;9(1):1557.
